# Enteric Fever Cases in the Two Largest Pediatric Hospitals of Bangladesh: 2013–2014

**DOI:** 10.1093/infdis/jiy521

**Published:** 2018-10-01

**Authors:** Shampa Saha, Mohammad J Uddin, Maksuda Islam, Rajib C Das, Denise Garrett, Samir Kumar Saha

**Affiliations:** 1 Child Health Research Foundation, Department of Microbiology, Dhaka Shishu (Children) Hospital, Bangladesh; 2 Sabin Vaccine Institute, Washington, DC; 3 Bangladesh Institute of Child Health, Dhaka Shishu (Children) Hospital, Bangladesh

**Keywords:** Bangladesh, enteric fever, hospital, *Salmonella*, surveillance

## Abstract

**Background:**

Enteric fever predominantly affects children in low- and middle-income countries. This study examines the burden of enteric fever at the 2 pediatric hospitals in Dhaka, Bangladesh and assesses their capacity for inclusion in a prospective cohort study to support enteric fever prevention and control.

**Methods:**

A descriptive study of enteric fever was conducted among children admitted in 2013–2014 to inpatient departments of Dhaka Shishu and Shishu Shashthya Foundation Hospitals, sentinel hospitals of the World Health Organization-supported Invasive Bacterial Vaccine Preventable Disease surveillance platform.

**Results:**

Of 15917 children with blood specimens received by laboratories, 2.8% (443 of 15917) were culture positive for significant bacterial growth. Sixty-three percent (279 of 443) of these isolates were confirmed as the cases of enteric fever (241 *Salmonella* Typhi and 38 *Salmonella* Paratyphi A). In addition, 1591 children had suspected enteric fever. Overall, 3.6% (1870 of 51923) were laboratory confirmed or suspected enteric fever cases (55% male, median age 2 years, 86% from Dhaka district, median hospital stay 5 days).

**Conclusions:**

The burden of enteric fever among inpatients at 2 pediatric hospitals in Dhaka, Bangladesh is substantial. Therefore, inclusion of these hospitals in a prospective cohort study will be useful for the generation of credible disease burden estimates of enteric fever in Bangladesh.

Enteric fever is an acute, systemic infectious disease caused by the bacteria *Salmonella enterica* serovars Typhi and Paratyphi A, B, or C, which often manifests with high-grade fever, coated tongue, nausea or vomiting, diarrhea, abdominal pain, and cough [1]. It predominantly affects children and young adults because they either lack natural immunity or experience high levels of exposure to fecal pathogens [2, 3]. Each year, approximately 16 million cases of illness and over 153000 deaths are attributed to enteric fever, although estimates vary and are uncertain due to the limited number of population-based incidence studies [4]. In addition, enteric fever continues to be an important global health problem especially in low- and middle-income countries of South Asia, including Bangladesh [2, 4–8]. These low-resource countries experience a high burden of enteric fever because they have limited access to safe drinking water and to adequate sanitation and hygiene [2, 9].

The establishment of a comprehensive surveillance system is important for generating credible data on enteric fever disease burden in endemic regions [10]. In Bangladesh, there was no comprehensive surveillance that systematically collected information on enteric fever for measuring burden of the disease. To date, the burden of enteric fever in Bangladesh has been estimated on the basis of studies conducted in an urban slum [11, 12]. Some hospital-based studies that estimated burden of enteric fever included hospitalized cases only [13, 14]. The lack of credible estimates for enteric fever incidence, complications, and mortality rates has hampered the establishment of evidence-based policy decisions to prevent and control this disease [15].

To fill these knowledge gaps, the Surveillance for Enteric fever in Asia Project (SEAP), a phased, comprehensive, multicountry, and multisite study, was initiated. The SEAP’s overall objectives are to characterize the burden of enteric fever in selected Asian settings, including clinical manifestations, severity of illness, long-term sequelae of illness, antimicrobial resistance patterns of *Salmonella* Typhi and Paratyphi, and cost of illness. The SEAP study will generate baseline data to inform appropriate interventions for enteric fever prevention and control and facilitate the assessment of the impact of interventions while also characterizing risk factors for the development of severe illness among enteric fever cases. Phase I of the SEAP was aimed at collecting data on enteric fever cases in 4 countries (Bangladesh, India, Pakistan, and Nepal) to assess health facilities with the potential to participate in and guide the design of Phase II of the project, a prospective surveillance study to be conducted at selected hospitals and laboratory networks in 3 endemic countries, namely, Bangladesh, Nepal, and Pakistan. In this study, we provide descriptive data from the SEAP Phase I to assess the burden of enteric fever at 2 pediatric hospitals in Dhaka, Bangladesh as well as evaluate their capacity to participate in a prospective cohort study.

## METHODS

### Study Design, Sites, and Procedures

Data for the SEAP Phase I were gathered retrospectively from an ongoing surveillance project during 2013–2014 among inpatient departments (IPDs) of 2 hospitals in Dhaka, Bangladesh: (1) Dhaka Shishu (Children) Hospital (DSH) and (2) Shishu Sashthya (Child Health) Foundation Hospital (SSFH). The DSH is the largest pediatric hospital in Bangladesh that provides primary, secondary, and tertiary care to patients less than 18 years of age; 37% of the 640 beds in DSH are reserved for patients who are unable to pay. With 200 beds, SSFH is the second largest pediatric (patients <18 years) hospital in the country and provides primary care only; 5% of admitted patients are provided care free of charge. In both hospitals, blood specimens are collected at the treating physician’s discretion and processed in the in-house microbiology laboratories.

These 2 hospitals are sentinel sites of the World Health Organization (WHO)-coordinated Invasive Bacterial Vaccine Preventable Disease (IB-VPD) surveillance. In our ongoing WHO-supported IB-VPD surveillance at DSH and SSFH, we collect detailed demographic, clinical, and laboratory information electronically on hospitalized suspected and laboratory-confirmed pneumonia, sepsis, meningitis, and enteric fever cases who are younger than 5 years. The inclusion criteria for enteric fever is ≥38.9°C for ≥3 days [6]. Minimal demographic and clinical information (for example, age, sex, diagnoses on admission and discharge, hospital duration, etc) of the hospitalized children who are not enrolled in IB-VPD surveillance are recorded in the hospital database. For the present study, we extracted demographic (age, sex, address), clinical (diagnoses, symptoms, duration of hospitalization, final outcome), and laboratory data of enteric fever cases from the IB-VPD surveillance and hospital databases.

### Case Definition

A suspected case of enteric fever was defined as a patient with a final clinical diagnosis of enteric fever without laboratory confirmation of *Salmonella* Typhi/Paratyphi infection through blood culture. This final diagnosis was based on physician’s assessment at the end of patient follow-up (ie, discharge/death/referral/left against medical advice). A laboratory-confirmed case of enteric fever was defined as a patient whose blood culture was positive for *Salmonella* Typhi/Paratyphi.

### Detection of *Salmonella* Typhi and *Salmonella* Paratyphi in Blood Samples

All blood cultures were performed using standard methods [16]. In briefly, 2–3 mL blood was obtained under aseptic condition and inoculated into Trypticase soy broth supplemented with sodium polyethanol sulphonate (0.25%) and isovitalex (1%). After incubation, blood culture bottles were subcultured on second, third, and fifth days of incubation. Standard biochemical tests and agglutination with *Salmonella* species and serovar specific antisera (Ramel; Thermo Fisher Scientific) were used to confirm *Salmonella* Typhi/Paratyphi isolates. To ensure the quality of the laboratories, we have both internal and external quality control (QC) systems in place. Internal QC of the laboratories is performed at quarterly basis. External QC is performed once a year by United Kingdom National External Quality Assessment Scheme as part of QC of IB-VPD surveillance. In addition, we send specimens to the Center for Disease Control and Prevention to determine whether the results were concordant. Blood culture volume is regularly monitored in the laboratories.

### Data Analysis

All analyses were performed using the STATA 13.1 (StataCorp LP, College Station, TX). Summary statistics included median ± interquartile range (IQR) for continuous variables and frequencies with percentages for categorical variables. Comparative statistics included independent samples *t* test, Wilcoxon’s rank-sum test, χ^2^ test, and Fisher’s exact test, as appropriate. Two-sided statistical tests were conducted at an alpha level of 0.05.

### Ethical Consideration

Approval for analysis of secondary data for SEAP Phase I was obtained from the Ethical Review Committee of Bangladesh Institute of Child Health, Dhaka, with a waiver of informed consent.

## RESULTS

### Enteric Fever Cases Identified at Each Site

**Figure 1.**
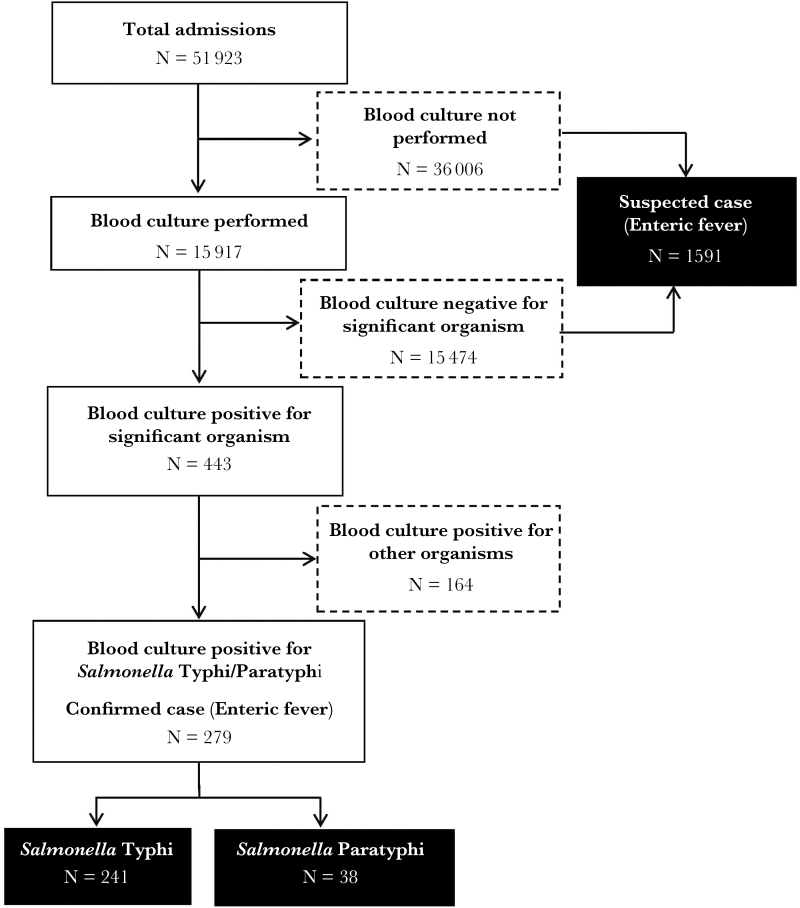
Flowchart for the identification of enteric fever cases at the study hospitals, Dhaka, Bangladesh, 2013–2014.

Between January 2013 and December 2014, a total of 51923 children were admitted in the IPDs of the 2 study hospitals (44111 at DSH and 7812 at SSFH) (Table 1 and Figure 1). The age of the hospitalized children ranged from 1 day to 18 years with a median of 7 months (IQR, <1 month–24 months). The majority of children (88.2%, 45790 of 51923) admitted to these hospitals were less than 5 years of age, with 73.9% (38364 of 51923) younger than 2 years.

**Table 1.** Total Admissions, Blood Culture Performed, and Enteric Fever Cases by Study Hospital, Dhaka, Bangladesh, 2013–2014 (n = 51923)

Parameters	Dhaka Shishu Hospital		Shishu Shasthya Foundation Hospital		Total	
	n	%	n	%	n	%
Total admissions	44111		7812		51923	
Blood culture performed	11888	27.0	4029	51.6	15917	30.7
Blood culture positive, any organism	289	2.4	154	3.8	443	2.8
Blood culture positive, enteric fever	151	1.3	128	3.2	279	1.8
*Salmonella* Typhi	135	89.4	106	82.8	241	86.4
*Salmonella* Paratyphi A	16	10.6	22	17.2	38	13.6

Of 51923 children admitted to these 2 hospitals, 15917 had blood specimens collected and received by laboratories for culture, and 2.8% (443 of 15917) of these specimens were culture positive for significant microbiological growth. The total number of patients eligible for blood draw was not available. Furthermore, 1.8% of all blood cultures (279 of 15917) or 63% of culture-positive cases (279 of 443) were laboratory-confirmed enteric fever cases; 86% (241 of 279) of them were laboratory-confirmed *Salmonella* Typhi, and 14% (38 of 279) were *Salmonella* Paratyphi A. Approximately 83% (128 of 154) of all isolates in SSFH were positive for *Salmonella* Typhi of Paratyphi A, whereas 52% (151 of 289) of all isolates in DSH were positive for *Salmonella* Typhi/Paratyphi A (Table 1 and Figure 1). Among hospitalized children who either had no blood culture done or whose blood culture was negative for microbiological growth (n = 51480), 1591 had a final clinical diagnosis of enteric fever.

In total, 3.6% (1870 of 51923) of all admissions at the study hospitals were either laboratory-confirmed or suspected enteric fever cases; the proportion of laboratory-confirmed and suspected enteric fever cases was significantly higher (*P* < .001) in SSFH (9.1%, 711 of 7812) than in DSH (2.6%, 1159 of 44111).

### Characteristics of Suspected and Laboratory-Confirmed Enteric Fever Cases

**Figure 2.**
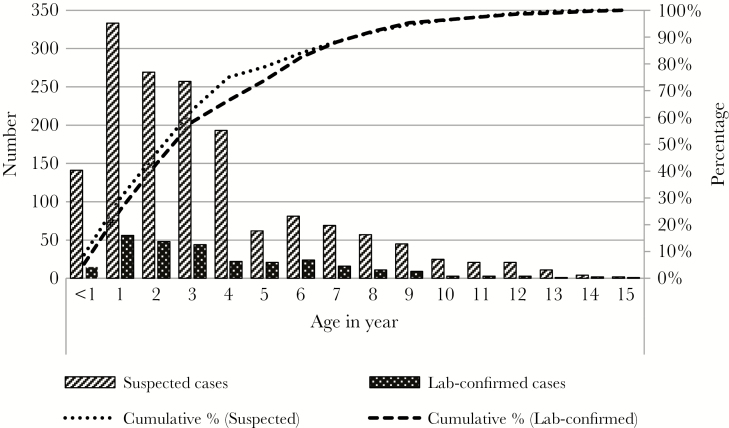
Age distribution of suspected and laboratory-confirmed enteric fever cases who were admitted at the study hospitals, Dhaka, Bangladesh, 2013–2014 (n = 1870).

Among all suspected and laboratory-confirmed enteric fever cases, 55% (1032 of 1870) were male. The majority (86%, 1606 of 1870) of cases lived within the Dhaka district. Detailed information on residential address was available for 61% (1133 of 1870) of cases. Of them, 83% (946 of 1133) lived within Dhaka; 55% (626 of 1133) lived within 30 minutes travel time to either of the hospitals, and 28% (320 of 1133) lived more than 30 minutes travel time away. Approximately three quarters of all suspected and laboratory-confirmed enteric fever cases (74%, 1193 of 1870) identified at the study hospitals were younger than 5 years, and 8% (156 of 1870) of enteric fever cases were infants (<1 year) (Figure 2). Among infants, 90% (141 of 156) were suspected and 10% (15 of 156) were laboratory-confirmed cases. No laboratory-confirmed enteric fever cases were identified among infants younger than 7 months. The median age of suspected and laboratory-confirmed enteric fever cases at both hospitals was 3 (IQR, 1–5) years. Compared with SSFH (median, 3; IQR, 2–6 years), admitted suspected and laboratory-confirmed enteric fever cases were younger at DSH (median, 2; IQR, 1–4 years; *P* < .001). Laboratory-confirmed cases were significantly more likely to live within the Dhaka area and had significantly longer duration of hospitalization compared with suspected cases of enteric fever (Table 2).

**Table 2.** Characteristics of Suspected and Laboratory-Confirmed Enteric Fever Cases, Dhaka, Bangladesh, 2013–2014 (n = 1870)

Characteristics	Suspected (n = 1591)		Confirmed (n = 279)		Total (n = 1870)		*P* Value
	n	%	n	%	n	%	
Age (median [IQR]), years	3 (1–5)		3 (1–6)		3 (1–5)		.05
Gender							
Female	711	44.7	127	45.5	838	44.8	.79
Male	880	55.3	152	54.5	1032	55.2	
Home Address							
Within Dhaka	1341	84.3	265	95.0	1606	85.9	<.001
Outside Dhaka	250	15.7	14	5.0	264	14.1	
Duration of hospitalization (median [IQR]), days	5 (4–7)		6 (4–8)		5 (4–7)		<.001
Outcome							
Discharged	1494	93.9	267	95.7	1761	94.2	
Left against medical advice	94	5.9	10	3.6	104	5.6	.003
Referred	0	0.0	2	0.7	2	0.1	
Died	3	0.2	0	0.0	3	0.2	

Abbreviation: IQR, interquartile range.

The majority (89%, 1658 of 1870) of all cases were clinically diagnosed with enteric fever on admission (Table 3). Other frequent diagnoses included gastrointestinal tract infection (n = 57, 3%) and lower respiratory infection (n = 38, 2%). Median hospital duration of the enteric fever cases was 5 (IQR, 4–7) days. Hospital duration for confirmed cases (median, 6; IQR, 4–8 days) was longer than that for suspected cases (median, 5; IQR, 4–7 days; *P* < .001). At DSH, enteric fever cases had longer hospital stay (median, 5; IQR, 4–7 days) compared with that at SSFH (median, 5; IQR, 3–6 days; *P* < .001). Three deaths occurred (0.2% of cases) and all were among suspected cases. Two deceased cases were infants, whereby no blood culture was performed, and the third case was a 3-year old child, whose blood culture did not yield any microbiological growth.

**Table 3.** Clinical Diagnoses of Suspected and Laboratory-Confirmed Enteric Fever Cases on Admission, Dhaka, Bangladesh, 2013–2014 (n = 1870)

Diagnosis on Admission	Suspected (n = 1591)		Confirmed (n = 279)		Total (n = 1870)		*P* Value
	n	%	n	%	n	%	
Enteric fever	1432	90.0	226	81.0	1658	88.7	<.001
Gastrointestinal tract infection	40	3.0	17	6.1	57	3.0	.002
Lower respiratory infection	33	2.0	5	1.8	38	2.0	.764
Febrile convulsion	13	1.0	14	5.0	27	1.4	<.001
Viral fever (including dengue fever and mumps)	15	1.0	2	0.7	17	0.9	.714
Urinary tract infection	12	1.0	1	0.4	13	0.7	.463
Central nervous system infection	5	0.0	7	2.5	12	0.6	<.001
Sepsis	9	1.0	0	0.0	9	0.5	-
Hepatitis	3	0.0	2	0.7	5	0.3	.115
Pyrexia of unknown origin	4	0.0	0	0.0	4	0.2	-
Upper respiratory infection	2	0.0	0	0.0	2	0.1	-
Other	23	1.4	5	1.8	28	1.5	.660

Information on the clinical profile of enteric fever cases was available for 834 (45%) cases, including 555 (35%) suspected and 279 (100%) confirmed cases. All of these cases had history of fever (100%), and almost all cases (n = 795, 95%) had high fever (temperature ≥38.9 °C) on the day of admission (Table 4).

**Table 4.** Clinical Profile of Suspected and Laboratory-Confirmed Enteric Fever Cases, Dhaka, Bangladesh, 2013–2014 (n = 834)

Variable	Suspected (n = 555)		Confirmed (n = 279)		Total (n = 834)		*P* Value
	n	%	n	%	n	%	
Symptoms							
History of fever	555	100.0	279	100.0	834	100.0	-
Diarrhea	155	27.9	98	35.1	253	30.3	.033
Vomiting	148	26.7	96	34.4	244	29.3	.02
Abdominal pain	42	7.6	45	16.1	87	10.4	.0001
Convulsion	31	5.6	20	7.2	51	6.1	.368
Constipation	23	4.1	8	2.9	31	3.7	.357
Abdominal tenderness	4	0.7	11	3.9	15	1.8	.002
Headache	5	0.9	0	0.0	5	0.6	-
Difficulty in breathing	9	1.6	0	0.0	9	1.1	-
Blood in stool	2	0.4	1	0.4	3	0.4	.739
Rash	3	0.5	0	0.0	3	0.4	-
Maximum Temperature on the Day of Admission (°C)							
Mean (standard deviation)	39.49 (0.60)		39.52 (0.69)		39.50 (0.63)		.238
≥38.9	399	71.89	212	75.99	611	73.26	
38–<38.9	147	26.49	56	20.07	203	24.34	
<38	9	1.62	11	3.94	20	2.40	

## DISCUSSION

Enteric fever remains a public health problem in middle- and low-income countries. In this study, we report that suspected or laboratory-confirmed enteric fever was responsible for approximately 4% of all hospitalizations at the 2 largest pediatric hospitals in Dhaka, Bangladesh between 2013 and 2014. We also report that over 60% of culture-positive cases had confirmed enteric fever and that *Salmonella* Typhi was the primary pathogen identified. Although 3 patients died during hospitalization, none of these deaths were reported among laboratory-confirmed typhoid or paratyphoid cases.

In general, our study finds that a large percentage of infectious diseases in Dhaka can be attributed to enteric fever, consistent with single-center studies previously conducted in Bangladesh [14, 17]. For instance, one study collected blood specimens from 103679 hospitalized and nonhospitalized patients attending the hospital attached to icddrb, between January 2005 and December 2014, and found that 13.6% of cultured blood samples were positive, with *Salmonella* Typhi being the most frequently isolated microorganism (36.9% of positive blood samples) [14].

Among the hospitalized enteric fever cases, the majority were younger than 5 years, which is consistent with earlier studies [6, 11, 13, 18]. The overall isolation rate of *Salmonella* Typhi and *Salmonella* Paratyphi A was lower than that reported by 3 studies [11–13]. This may be because the surveillance was not planned to systematically identify enteric fever cases in all pediatric hospitalized patients. The proportion of laboratory-confirmed cases was lower in children who lived outside Dhaka compared to that in the children that lived within Dhaka. This is possibly due to the fact that the cases who come from outside Dhaka are usually referred from other hospitals and thereby treated with antibiotic before referral. It is likely that this prior treatment with antibiotic reduced the chance of yielding a positive growth in blood culture.

This study is not without limitations. Reliance on existing hospital surveillance databases may lead to missing data on key clinical characteristics. Moreover, the study only collected information of cases that were hospitalized at pediatric medicine IPDs. Many enteric fever cases receive treatment at outpatient departments (OPD) and are generally not hospitalized (Saha S et al, submitted). Some cases might have been admitted to the surgery department for complications of enteric fever and were not included in the ongoing surveillance. Inclusion of enteric fever cases that were treated at the OPD or other departments is likely to provide a more comprehensive overview of the spectrum of the disease. The SEAP Phase II is specifically designed for identification of enteric fever and is expected to address these limitations by estimating the burden and severity of enteric fever through comprehensive enteric fever surveillance.

## CONCLUSIONS

Despite all of these limitations, our study demonstrated high burden of culture-confirmed enteric fever (*Salmonella* Typhi or *Salmonella* Paratyphi A) at IPDs of 2 pediatric hospitals (DSH and SSFH) in Dhaka, Bangladesh. Enrollment of cases from diverse departments (OPD and IPD) of these 2 hospitals will likely enhance the generalizability of enteric fever incidence calculations and make the SEAP Phase II a success.

## Notes

***Financial support.*** This work was supported by a grant from the Bill and Melinda Gates Foundation (OPP11130007) and the World Health Organization (201588766, 201233523, 201022732, 200749550). This article is part of the supplement “Surveillance of Enteric Fever in Asia Project,” sponsored by the Sabin Vaccine Institute.

***Supplement sponsorship.*** This article is part of the supplement “Surveillance for Enteric Fever in Asia Project,” sponsored by the Sabin Vaccine Institute.

***Potential conflicts of interest.*** S. K. S. has received research grants from GlaxoSmithKline, Sanofi Pasteur, and Pfizer. All authors have submitted the ICMJE Form for Disclosure of Potential Conflicts of Interest. Conflicts that the editors consider relevant to the content of the manuscript have been disclosed.
